# Bioinformatics Study on Site-Specific Variations of Eotaxin-3, a Key Chemokine in Eosinophilic Esophagitis (EoE)

**DOI:** 10.3390/genes15081073

**Published:** 2024-08-14

**Authors:** Deborah Giordano, Antonio d’Acierno, Anna Marabotti, Paola Iovino, Giuseppe Iacomino, Angelo Facchiano

**Affiliations:** 1 National Research Council, Institute of Food Science, 83100 Avellino, Italyangelo.facchiano@isa.cnr.it (A.F.); 2 Department of Chemistry and Biology “A. Zambelli”, University of Salerno, 84084 Fisciano, Italy; 3 Department of Medicine, Surgery and Dentistry “Scuola Medica Salernitana”, University of Salerno, 84081 Baronissi, Italy

**Keywords:** eosinophilic esophagitis, protein variants, protein structural analysis, protein modelling, web application

## Abstract

Eotaxin-3 is a key chemokine with a relevant role in eosinophilic esophagitis, a rare chronic immune/antigen-mediated inflammatory disorder. Eotaxin-3 is a potent activator of eosinophil emergence and migration, which may lead to allergic airway inflammation. We investigated, using bioinformatics tools, the protein structure and the possible effects of the known variations reported in public databases. Following a procedure already established, we created a 3D model of the whole protein and modeled the structure of 105 protein variants due to known point mutations. The effects of the amino acid substitution at the level of impact on protein structure, stability, and possibly function were detected by the bioinformatics procedure and described in detail. A web application was implemented to browse the results of the analysis and visualize the 3D models, with the opportunity of downloading the models and analyzing them using their own software. Among 105 amino acid substitutions investigated, the study evidenced in 44 cases at least one change in any of the investigated structural parameters. Other six variations are also relevant, although a structural effect was not detected by our analysis, because they affected amino acids highly conserved, which suggests a possible function role. All these variations should be the object of particular attention, as they may induce a loss of functionality in the protein.

## 1. Introduction

Eosinophilic gastroenteritis (EGE) is a rare chronic immune/antigen-mediated inflammatory disorder. EGE has historically been described as an eosinophilic disease involving more than one gastrointestinal tract, including the esophagus in 77% of cases. Recently, an international consensus has systematized the various nosographic entities and brought them together under the general term eosinophilic gastrointestinal diseases (EGID) due to the disorder’s assorted clinical presentation [1]. EGID are conditions characterized by an excessive infiltration of eosinophils, which can affect any part of the digestive tract. Specific forms of EGID include eosinophilic gastritis, eosinophilic gastroenteritis, eosinophilic esophagitis (EoE), and eosinophilic colitis, depending on the anatomical tracts involved.

EoE is a chronic inflammatory condition of the esophagus commonly associated with symptoms of esophageal dysfunction. This disease is primarily caused by abnormal eosinophil infiltration in the esophageal mucosa, along with the contribution of several other types of inflammatory mediators [2]. EoE is considered the most common EGID. The estimated incidence in adults and children is 7.0 and 5.1 per 100,000/year, respectively, with an overall prevalence estimate of 34 per 100,000/year [3]. The disease is more common in males across all age groups, with a male-to-female ratio of 3:1. The peak incidence occurs between ages 20 and 40 years, with up to 22% of patients undergoing upper endoscopy for non-obstructive dysphagia and more than 50% of patients referred for food bolus impaction receiving a diagnosis of EoE. Yet, EoE symptoms have a significant impact on quality of life, often causing psychological distress. While EoE was initially thought to be rare, its incidence and prevalence have been rapidly increasing in both children and adults worldwide. This increase prompts the question of whether EoE is truly a new disease or simply a newly recognized condition. Moreover, there is a relevant epidemiological question about whether the rise in EoE cases is due to the increased allergenicity of foods or the increased susceptibility of individuals to environmental factors. Notably, there has been a significant global increase in the prevalence of allergies in recent decades. Accordingly, most diagnosed adult patients have comorbid conditions allergic rhinitis, asthma, IgE-mediated food allergies, and atopic dermatitis [4,5], while higher rates of hypersensitivity to food antigens are found in children.

EoE diagnosis is based on three main parameters that are common across all age groups. These include symptoms of esophageal dysfunction, the presence of at least 15 eosinophils per High Power Field (HPF) or 60 eosinophils per mm^2^ on biopsies taken during endoscopy, and the exclusion of other non-EoE disorders that may cause or contribute to esophageal eosinophilia. These disorders include eosinophilic gastritis, gastroenteritis or colitis with esophageal involvement, gastroesophageal reflux disease (GERD), achalasia and other esophageal motility disorders, hypereosinophilic syndrome, Crohn’s disease with esophageal involvement, fungal or viral infections, connective tissue disorders, hypermobility syndromes, autoimmune disorders and vasculitis, dermatologic conditions with esophageal involvement (e.g., pemphigus), drug hypersensitivity reactions, pill-induced esophagitis, “graft-versus-host disease,” and Mendelian disorders. It is worth noting that only about 30% of cases show increased eosinophils in peripheral blood in laboratory tests.

EoE pathogenesis involves a complex interaction of genetic and environmental factors, including diet and antigenic load, but a full understanding remains elusive [2], with evidence suggesting that allergies play a significant role in EoE. First, special diets that remove certain foods often help. Second, EoE often occurs with other allergies, such as eczema and asthma. Third, researchers can cause EoE in animals by exposing them to allergens. Finally, molecules involved in allergies, such as IL-5 and IL-13, seem required for the inflammation in EoE.

Twin studies indicate that the etiology of EoE is also influenced by genetic factors, with a higher disease concordance observed in monozygotic twins compared to dizygotic twins. Overall, EoE is believed to be influenced by a combination of genetic factors, with known risk variations having a modest impact on the overall risk of the disease. The risk variants associated with EoE patients are supposed to primarily influence the regulation of gene expression, and hundreds of genes show altered levels in the esophagus of affected individuals, suggesting that the disorder has a complex genetic component. Furthermore, the significant variations in IL-13 in esophageal cells strongly suggest that this cytokine plays a crucial role in regulating genes associated with disease development [6].

In genome-wide association studies, genetic variants at three loci (5q22 [TSLP/WDR36], 2p23 [CAPN14], and 11q13 [LRRC32/EMSY]) have consistently been identified. In other studies, significant associations were found at 12q13 (STAT6), 19q13 (ANKRD27), and 16p13 (CLEC16A). Other studies have identified PTEN, TGFBR1/TGFBR2/PBN, and IL5/IL13 as crucial risk loci for EoE. Finally, evidence from epidemiologic studies strongly suggests that EoE has a substantial genetic component, likely as a result of gene-environment interactions, particularly those involving early-life exposures [7].

A key EoE chemokine for eosinophil chemotaxis is eotaxin-3 (CCL26; C-C Motif Chemokine Ligand 26), a potent activator of eosinophil emergence and migration, potentially triggering allergic airway inflammation. Its ability to attract eosinophils is stronger as compared to other chemokines [8], representing one of the most highly induced genes in the esophageal epithelium and peripheral blood during active inflammation in EoE patients [9,10]. Similar to EoE, epithelial cells from both healthy individuals and GERD patients express eotaxin-3 upon stimulation by T_H_2 cytokines [11], thus embodying the role of a critical driver of eosinophil migration to the esophageal tissue. Located on chromosome 7q, the CCL26 gene encodes a protein produced by vascular endothelial and lung epithelial cells upon stimulation by IL-4 or IL-13. Eotaxin-3 belongs to a group of three chemokines that activate the CCR3 receptor, leading to calcium mobilization and the recruitment of eosinophils and basophils from peripheral blood to inflammation sites [12]. Remarkably, eotaxin-3 also exhibits antimicrobial activity. In atopic diseases, it likely contributes to eosinophil accumulation. Additionally, research suggests a role for eotaxin-3 in promoting epithelial-mesenchymal transition and even tumor growth and invasion [13].

According to information available in public databases (see below under Section 2), eotaxin-3 is subjected to point variations that may alter the correct function of this protein. In the past years, we have established a bioinformatics procedure to generate an integrated database and a web application to gather and distribute information regarding predicted structural and functional impacts of genetic variations on several proteins [14], and possibly to assess their connection with the clinical outcomes of the related diseases. We freely shared these results with the scientific community through our web platform [14]. This allowed researchers worldwide to access relevant information for their specific research. We decided to develop a new web application that will also gather relevant information about eotaxin-3 and the potential effects of its known amino acid substitutions to contribute to the understanding of the protein structure and function and possible impairment due to its single nucleotide variation with consequent amino acid substitution. We investigated the eotaxin-3 point variations listed in the UniProt database for the possible effects on protein structure and stability by integrating bioinformatics analysis of the wild-type protein structure, molecular modelling of the single amino acid substitutions and evaluation of the effects in comparison to the wild-type protein. The results are described in this article and available via a web interface for free access to any interested research team. The potential applications include the possible interest in specific variations as potential markers of disease conditions.

## 2. Materials and Methods

### 2.1. Protein Model Construction

General information for the eotaxin-3 sequence and its variations was retrieved by the UniProt database [15], where eotaxin-3 is reported with UniProt code Q9Y258. The PDB database [16] reports only two NMR structures (PDB code: 1G2S and 1G2T), both, as expected, lacking the first 23 residues corresponding to the signal peptide. A complete AlphaFold model [17] is instead available (identifier: AF-Q9Y258-F1), although with a low confidence score for the N- and C- terminal parts of the protein. Due to the lower quality of the AlphaFold model compared to the PDB structure from NMR data and the incompleteness of this latter, we decided to build by Modeller 9.22 [18] novel ten 3D models exploiting as templates both the structures, the NMR one and the AlphaFold prediction. PDB structure with code 1G2S was preferred to one of the 20 NMR models present in the PDB file 1G2T because it represents their energy-minimized average structure [19]. Models were assessed by ProSA-web [20] for the calculation of the Z-score, ProCheck [21] for the analysis of the Ramachandran plot provided by the SAVES v6.0 platform, Q-MEAN provided by SWISS-MODEL [22], and the RMSD calculated by PyMol [23] and using 1G2S as a reference structure. Among the ten models built, the one that exhibited the best quality parameters was chosen as the reference 3D model for the wild-type protein and has been used as the base for the variant model construction by Modeller 9.22, exploiting the python script mutate_model.py (available from the Modeller web site: https://salilab.org/modeller/wiki/Mutate_model, accessed on 31 July 2024). Graphical representations of the protein have been obtained by Discovery Studio (Dassault Systèmes) and Ligplus [https://www.ebi.ac.uk/thornton-srv/software/LigPlus/, accessed on 31 July 2024].

Modeled variants are all the missense mutations available for each protein in the UniProt database in the disease and variants section (https://www.uniprot.org, accessed on 24 June 2024). In particular, 105 mutations were modeled and analyzed for eotaxin-3.

### 2.2. Prediction of the Variant Impact

Once the mutant models had been obtained, the impact of these variants on the structure and function of exotoxin-3 was predicted using an automated procedure for performing a series of structural analyses, as in previous and similar work [14,24]. More in-depth wild-type and mutant models were analyzed, and the results obtained were compared by DSSP [25] to investigate variation in the secondary structure, by HBPLUS [26] to highlight the difference in terms of H-bond formation, and by NACCESS [27] to detect the change in the solvent accessibility of each residue object of variation. Furthermore, a script generated in-house allows the detection and comparison of the salt bridge between positively and negatively charged residues. In addition, the effect of these mutations on the stability of the protein was analyzed using a consensus method, which included the results of five stability predictors releasing the one achieved by the majority. The five different web servers are MAESTROweb [28], INPS-3D [29], PoPMuSiC [30], DynaMut2 [31], and DUET [32]. When at least 3 out of 5 predictors gave the same result overcoming the cutoff of significance of each method [33], the mutation was classified as “more stable” or “less stable”, while it was classified as “uncertain” when the consensus of the predictors was not achieved.

### 2.3. Database and Web Interface

The results of all the analyses made on wild-type and mutant models were stored in a database, freely accessible through a web interface accessible at the following URL: http://www.protein-variants.eu/eotaxin-3-protein-db/, accessed on 31 July 2024. In addition, the database collects information about the nucleotide substitution determining the variation (from the UniProt entry), exon organization (from NCBI Reference Sequence entry: NG_015989.1), and the amino acid conservation score (calculated by the Consurf server [34]). The web application is a generalization of the tool described in our previous works [14,24].

## 3. Results and Discussion

### 3.1. Protein Modelling of Human Eotaxin-3 and Its Variants

**Figure 1:**
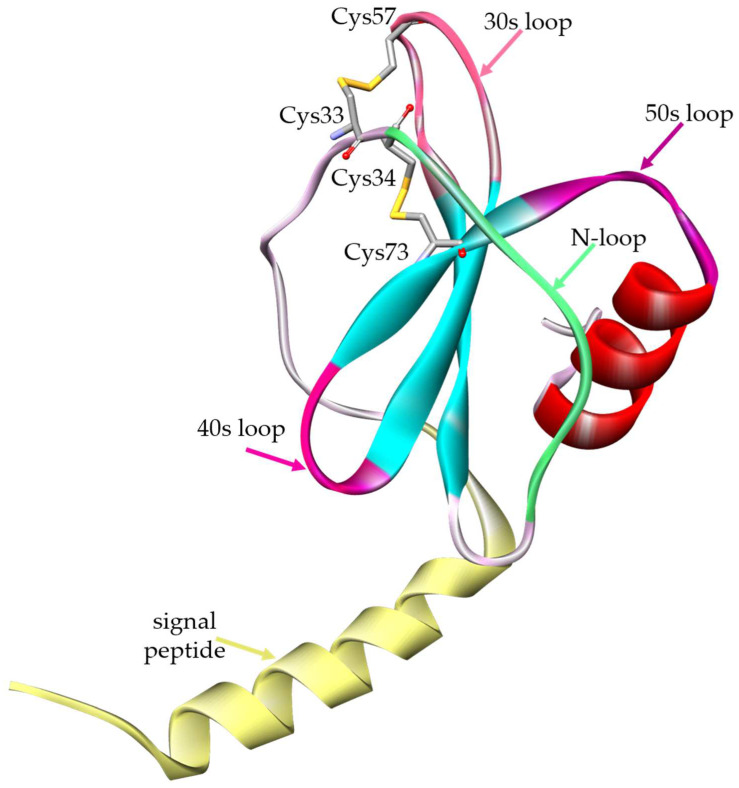
3D model of eotaxin-3 in the pre-cleaved form. The image shows the backbone of the complete sequence, represented as a ribbon. The N-terminal signal peptide (yellow) is followed by the region with an N-loop (green), a central β-sheet whose β-strands (cyan) are connected by three turns (30s, 40s, 50s loop), and a C-terminal α-helix (red). Two disulfide bonds occur between C33-C57 and C34-C73 (shown in stick representation). Image has been generated by Discovery Studio software.

The study of human eotaxin-3 available structures found on RCSB PDB highlighted the presence of NMR eotaxin-3 structures only in the mature form of the protein, thus missing the first 23 residues in the N-terminal region corresponding to the signal peptide required for protein secretion. However, for a full investigation of possible alteration in protein secretion due to mutations in this region, a model of the whole eotaxin-3 precleaved form was needed. Therefore, in order to obtain a complete 3D structure of the protein, a full chain model of the pre-cleaved form was created, based on the structural model identified by the PDB code 1G2S and the predicted AlphaFold model: AF-Q9Y258-F1. The model obtained (Figure 1) also includes the signal peptide region, which is not present in the NMR model, and offers, as a benefit over the AlphaFold model, quality improvement in terms of energetic and stereochemical properties (see Table 1).

The resulting model of the pre-cleaved form of the wild-type eotaxin-3 was used to generate in silico the 3D models of the missense variants of the protein selected as described in the next paragraph.

Models of the variants were then analyzed and compared to the wild-type in order to obtain information on the variants’ impact in terms of structure, stability, and possibly protein functionality. The data obtained were collected in the free database available at http://www.protein-variants.eu/eotaxin-3-protein-db/, accessed on 31 July 2024. For each variant, the database provides a detailed analysis of the structural parameters detectable in the mutated amino acid and compared with the wild-type protein using convenient side-by-side tables. Each variant can be viewed on the web application via a 3D viewer, just as the user has the option of downloading the 3D model in PDB format and analyzing it with their own bioinformatics tools.

In the following paragraphs, we describe the selection of the protein variants and the predicted effects of the main variations.

### 3.2. Human Eotaxin-3 Variants Selection and Mapping

**Figure 2:**
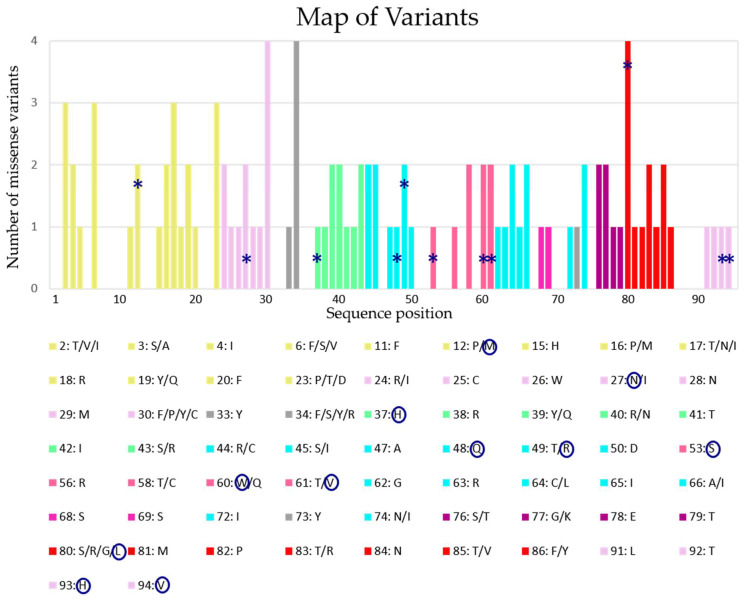
Protein sequence map of the 105 variants analyzed. The plot represents a map of the number and the position of the 105 variants analyzed along the sequence. On the x-axis is reported the position of each amino-acidic residue composing eotaxin-3, and on the y-axis is the number of missense mutations that occur in that specific position; the detail about each amino-acidic substitution in its particular position is reported in the legend. Bars are colored according to residue position in the 3D model, reported in Figure 1. The missense variants annotated as having moderate impact in UniProt are highlighted with a star (*) on the bars and circled in the legend.

The selection of human eotaxin-3 variants started with the analysis of the protein entry Q9Y258 in the UniProt database variant table. The table collects information about variants from different databases. The whole number of variants, i.e., 115, includes frameshifts and stop codons that induce strong modifications in the protein structure and are not analyzed in our approach, which aimed to investigate the subtle effects of amino acid substitution. The variants caused by amino acid substitution are 105, and their mapping on the sequence is shown in Figure 2. They were selected for the analysis of their impact on the protein structure and stability.

The position of the amino acid variations spreads along the entire sequence, signal-peptide included. In particular, 25 mutations map on the signal peptide, 10 in the N-loop, eight in the 30s loop, two in the 40s loop, six in the 50s loop, 20 in the central β-strands, 13 in the C-terminal α-helix, and 21 in the remaining portions of the structure. Among them, the UniProt database provides an automatic annotation only for 11 variants that, differently from the others, are derived from studies performed by the NCI-TCGA program. These 11 variants report the annotation of the moderate impact category assigned (Figure 2), and among them, three have been detected in patients with colon carcinoma (Lys12Met, Lys94Val) and lung cancer (Thr53Ser) and included in the COSMIC database. The annotation “moderate” is a predicted Sequencing Ontology (SO) type of the variants that categorized the impact of the SNP that is also compatible with snpEff. In particular, it is used to refer to a non-disruptive variant that might change protein effectiveness (inframe insertion/deletion, missense variant, protein altering variant, regulatory region ablation). This annotation differs from the SO “high” or “low” used predominantly for stop codon/frameshift variants or synonymous variants, respectively. There is no specific clusterization on a particular region of these mutations that spread along all the sequence, even if in the 30s loop there is the major frequency of localization in the same area, with three mutations annotated as moderate (Thr53Ser, Arg60Trp, Ala61Val). It is important to underline that several studies on other chemokines have identified that the dimer interface involves numerous intersubunit contacts between the N-terminus, N-loop, 30s loop, and residues in β3 [35]. Therefore, mutations in these areas could have a strong impact on protein interactions.

### 3.3. Overview of the Effects of Amino Acid Variations on Eotaxin-3

The 105 eotaxin-3 variants analyzed evidenced in 44 cases at least one change in any of the investigated structural parameters. Among them, 18 cases affect an amino acid with a conservation score equal to 7 or better, suggesting an important role for that residue at the structural or functional level. Moreover, further six variants affect an amino acid with a conservation score equal to 7 or better, although no structurally relevant change is observed by our analysis.

The overall variants analyzed in this work indicate that the secondary structure of the protein is affected only in three cases, the solvent accessibility in ten, while the salt bridges are modified with a gain only in one case. The H-bond interactions vary in 30 cases, while the predicted protein stability decreases in 22 cases. The complete summary of the impact for each variant at the level of the analyzed properties (i.e., secondary structure, solvent accessibility, stability, H-bonds, salt bridges) is reported in Supplementary Table S1.

The loss or gain of interactions such as H-bonds or salt bridges can lead to a change in protein stability, and in our analysis, there are 14 cases in which the loss of stability can be related to the loss of interactions. However, there are eight mutations predicted to affect the protein stability without any detectable effect on the other parameters investigated. In further paragraphs, some of these cases are analyzed in detail.

### 3.4. Effects on Disulfide Bonds, Secondary Structure, Salt Bridges, and H-Bonds

Eotaxin-3 is characterized by the typical Greek key structure present in all the chemokines, stabilized by two disulfide bonds. In particular, the immature protein starts with a signal peptide composed of 23 amino acids, after which there is an N-loop that will compose the N-terminal portion of the mature form; here the first two Cys are located close together (Cys33 and Cys34). A β sheet, composed of three strands connected by two turns called 30s and 40s, follows the N-terminal portion; a final helix closes the structure. The third Cys (Cys57) is located in the 30s loop, while the fourth Cys (Cys73) is located at the end of the third strand in the third loop connecting the last strand to the helix, named the 50s loop. In the case of eotaxin-3, the two disulfide bonds occur between Cys33-Cys57 and Cys34-Cys73. The preservation of this structure has a crucial role in the correct recognition of the chemokines by the specific receptors [36]. In our analysis, one variation substitutes Cys33, four substitute Cys34, and one substitutes Cys73, resulting in all six cases in the loss of a disulfide bond, with a potential destabilizing effect on the protein stability. On the other side, five amino acid substitutions add a new Cys in the protein, with a potential effect on the protein’s stability in terms of the opportunity of creating unexpected disulfide bonds, with an effect similar to the “ruffled” conformation of Anfinsen’s experiment. Two of these five variants do not evidence any other effect on the other parameters investigated.

Only three mutations affect the secondary structure and address two residues located one after another, i.e., Leu15 and Leu16. In the details, Leu15His, Leu16Pro, and Leu16Met affect the helix constituting the signal peptide that, in physiological conditions, is cleaved during its secretion from the cell, releasing the mature form of the protein. Perturbation of the correct conformation of this signal peptide could lead to a defect in protein activation and secretion, resulting in a protein lacking its correct function.

Despite the low number of mutations that seem to affect the secondary structure, noteworthy are also the variants that insert proline in place of other residues in a portion of the structure interested by turns or loops (Ala23Pro, Ser30Pro). Actually, the backbone portion of the proline leads to a reduction of the conformational space for phi-psi angles and may induce a distortion of the secondary structure. Due to the particular nature of this protein, its turns and loops orientation is crucial for its function. Moreover, Ala23Pro is the last amino acid composing the signal peptide; its altered orientation may also result in a defect in the cleavage of the mature form. The mutations Leu12Pro and Gln82Pro instead, although located in two α-helices, do not destroy the secondary structure, but it cannot be excluded that the alteration that they induce in the helices, due to the loss of the backbone H-bond, may destabilize the helix conformation and could have an impact on the protein function.

**Figure 3:**
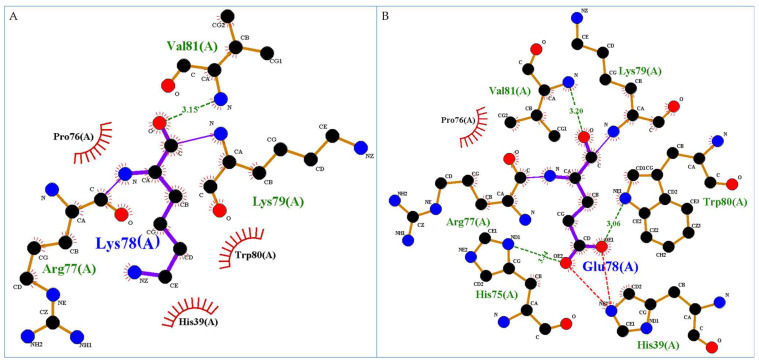
Detail of the interactions formed by the wild-type residue Lys78 (**A**) and the mutated residue Glu78 (**B**). Green dotted lines correspond to H-bond interactions, violet lines to the peptide bonds of the protein backbone, and the red dotted lines to the salt bridge, while residues shown as red arcs are involved in hydrophobic interactions. Images are generated by Ligplus v.2.2 software.

One mutation, Lys78Glu, seems to constitute a very significant change because it replaces a positive charge with a negative one. In particular, Lys78 in the wild-type form is located in the 50 s loop and creates an H-bond with Val81. The substitution of Lys by Glu determines the formation of two novel H-bonds and one salt bridge. In particular, the mutation Lys78Glu may make H-bonds with His75, Trp80, and Val81 and create a salt bridge with H39 that in the wild-type makes no interactions. In details, Val81 interactions remain unchanged, His75 (ND1 atom) gains an interaction with Glu78 (OE2 atom), while in the wild-type it has no interaction, Trp80 does not change its interactions with Lys83 and Tyr84 but gains an H-bond between its NE1 atom and OE1 atom of Glu78 (see Figure 3).

The acquisition of novel bonds by amino acids previously not directly involved in the intra-chain network is not always an improvement for the protein; actually, these amino acids could be involved in interactions with other proteins or with the CXC chemokine receptors, and their engagement in other bonds could make them less available for functional inter-chain interactions.

The most altered parameter is the H-bond interaction, with a loss or gain in 30 variations, and in 14 cases there is also a loss in terms of protein stability due to the general contribution of H-bonds to the conformational stability.

### 3.5. Variations Affecting Protein Stability and Function

**Figure 4:**
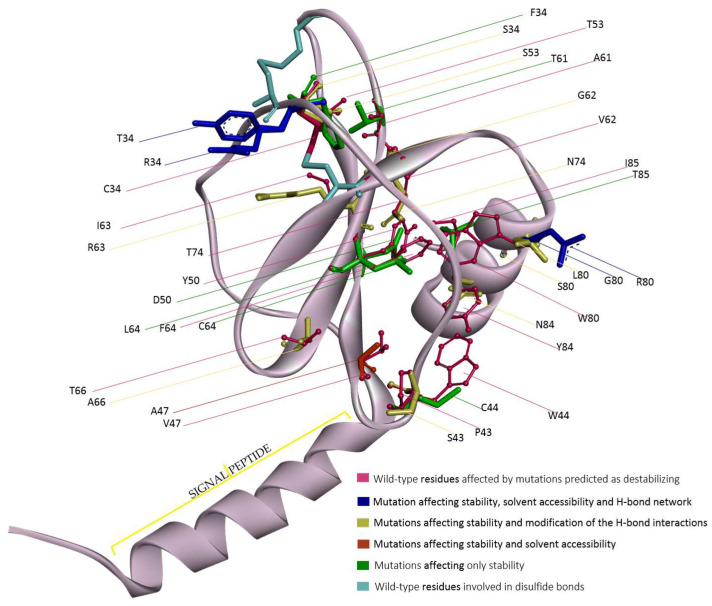
Structural position in the eotaxin-3 model of wild-type residues affected by mutations predicted as destabilizing and visualization of their mutations. Affected residues are shown as magenta ball and stick models. In overlap, the mutated residues are represented by sticks colored as reported in the graphical legend. The disulfide bonds are highlighted in cyan sticks. The image has been generated by Discovery Studio software.

There are 22 variants that are less stable than the wild-type protein. Among them, seven (Cys34Phe, Trp44Cys, Tyr50Asp, Ala61Thr, Phe64Cys/Leu, Ile85Thr) affect only protein stability without apparent effects on the other properties studied; ten (Cys34Ser, Pro43Ser, Thr53Ser, Val62Gly, Ile63Arg, Thr66Ala, Thr74Asn, Trp80Ser/Leu, Tyr84Asn) affect protein stability and vary their H-bond interactions; one (Val47Ala) affects only stability and solvent accessibility; while four (Cys34Tyr/Arg, Trp80Gly/Arg) affect solvent accessibility, H-bond interactions, and protein stability (Figure 4). Fourteen of these 22 variants involve buried residues; Tyr50, Trp84, and Ile85 are instead partially exposed residues, while Trp44 and Pro43 are totally exposed. Cys34Thr/Arg and Trp80Gly/Arg substitute a buried and a partially exposed residue, respectively, with totally exposed residues, while Val47Ala induces a change from buried to partially exposed.

In the case of all Cys34 mutations, the impact on protein stability can be due to the breaking of one of the two disulfide bonds. The other mutations instead map at the interface among the β-sheet and the C-terminal α helix, except for Pro43, Thr53, Ala61, and Thr66. Seven variants involve the substitution of polar residues with polar ones and three from polar, not charged residues to charged ones. The possible effect on stability is therefore explained also for those variants that seem not to affect the other properties analyzed. The change in polarity, together with the substitution of several aromatic residues at the interface by residues smaller and strongly different in shape, can lead to a possible loss of buried hydrophobic interaction with a possible effect on stability and consequent subtle structural rearrangements. Structural preservation is in fact fundamental for eotaxin-3 recognition by the CCR3 receptor. Moreover, studies of site-directed mutagenesis have indicated that the N-loop region, i.e., the loop following the second cysteine of chemokines, the 40s loop, and the starting portion of the β3 are important for receptor binding; therefore, mutation on Pro43 and Thr66 could alter CCR3 binding [19].

By comparing the wild-type model structure predicted by us and the crystallographic structure of the chemokine 20 in complex with its receptor (PDB code: 6WWZ) and by the alignment of their sequences, it was possible to detect that some residues affected by mutation may be at the protein-protein interface. Chemokine 20 forms several H-bonds with its receptor that involve Ser2 corresponding to Ile29 in eotaxin-3, Asn3 corresponding to Ser30, Cys48 to Cys73, Ala1 and Asp33 corresponding to Asp28 and Ser58, respectively (see Supplementary Figure S1 for the sequence alignment). All these eotaxin-3 residues are subject to mutations (Asp28Asn, Ile29Met, Ser30Pro, and Ser58Thr/Leu), for which no effect is evident on protein structure, protein stability, or intra-chain interactions, while Ser30Phe/Tyr/Cys and Cys73Tyr modify the solvent accessibility. However, their variation could have a functional effect by altering the correct interaction with the receptor. In a similar way, comparisons between eotaxin-3 and receptor-CCL7/15/2 complexes (PDB codes: 8JPS, 7VL9, and 7XA3, respectively) highlight key residues apparently not affecting protein properties. Specifically, from the comparison with the CCL7-receptor complex and eotaxin-3, Cys33Tyr, Lys40Arg/Asn, and Cys73Tyr impact residues involved in H-bonds and salt bridges in the crystallographic complex. From the comparison with CCL15 and CCL2 bound with their receptors, the mutations Asp28Asn, Cys33Tyr, Ser58Thr/Leu, and Arg25Cys, Gly26Trp, respectively, could alter the H-bond interactions of eotaxin-3 with a similar receptor. Noteworthy residue in position 30 in eotaxin-3 is always involved in H-bonds with the receptors in all the chemokine-receptor complexes analyzed; thus, mutations on Ser30 of eotaxin-3 suggest a possible impact on the binding to its specific receptor. However, this analysis of the possible alterations of the protein-protein interface, although offering indications useful to detect effects of the variations, is based on protein-receptor complex structures of chemokines with low degree of sequence similarity with eotaxin-3, even if very similar for their 3D structure (see Supplementary Figure S1).

**Figure 5:**
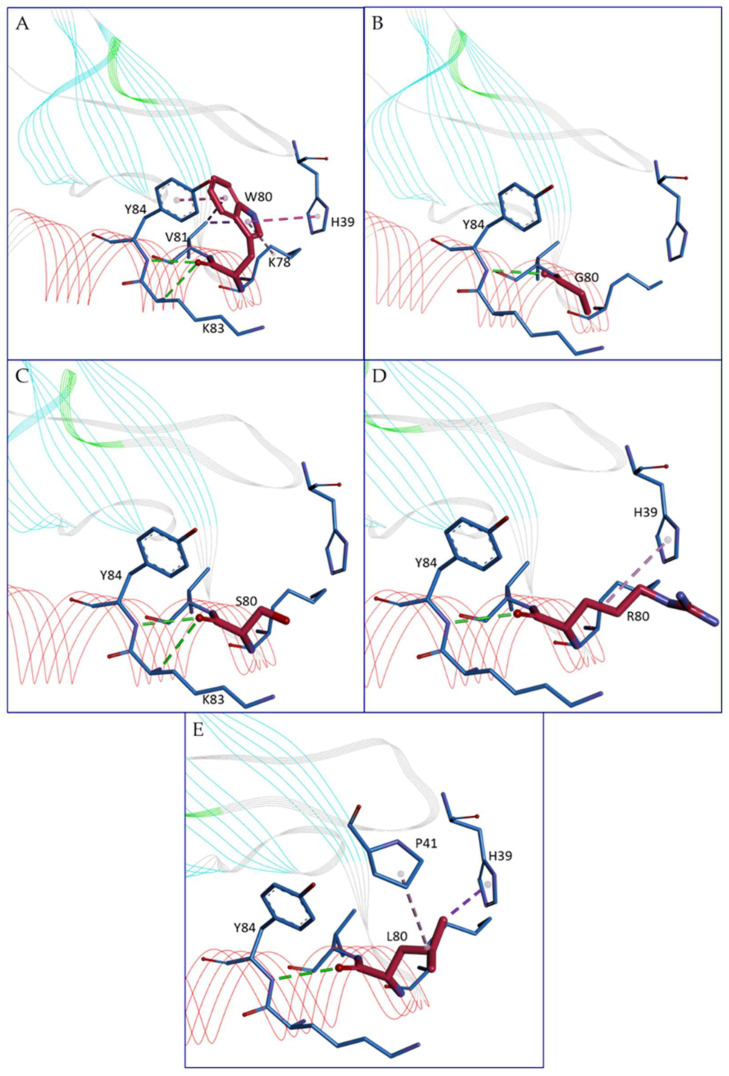
Details of the intra-chain interactions formed by Trp80 (**A**) or by its substituents, i.e., Gly80 (**B**), Ser80 (**C**), Asp80 (**D**), Leu80, (**E**). Residue 80 is always represented in magenta sticks, while the residues interacting with W80 in the wild-type are shown in blue sticks. Residues interacting with residue 80 are labeled. Hydrophobic interactions are shown as purple dotted lines, while H-bond interactions are visible as green dotted lines. Protein is represented in a line ribbon and colored by secondary structure. Image has been generated by Discovery Studio software.

Among the 105 missense variants analyzed, 11 show the annotation of the moderate impact category assigned by NCI-TCGA (Leu12Met, Ser27Asn, Tyr37His, Arg48Gln, Ser49Arg, Thr53Ser, Arg60Trp, Ala61Val, Trp80Leu, Gln93His, and Leu94Val), which indicates them as non-disruptive variants although they might affect protein functionality. Among these mutations, the ones for which our analyses reveal structural effects are Arg48Gln, Ser49Arg, Thr53Ser, and Trp80Leu. In more detail, Arg48Gln seems to induce a change in solvent accessibility, raising the exposition to the solvent. Ser49Arg modifies the H-bond network by creating additional H-bonds with Tyr84 and Ile85. Thr53Ser causes a loss of stability and perturbation of the H-bond interactions. Moreover, it is a highly conserved residue together with Ala61 and Trp80. Trp80, located at the beginning of the C-terminal helix, seems to be a crucial, highly conserved residue. It is the object of four mutations, and its change has a strong impact on several features. In the wild-type, Trp80 makes hydrophobic interactions with Val81, His39, Tyr84, and Lys78 and can make an H-bond with Lys83 or Tyr84, respectively (Figure 5A). Its mutation into Gly raises the relative solvent accessibility and destroys the possible H-bond with Lys83 and all the hydrophobic interactions, losing all the interactions between the C-terminal helix and the N-loop region (Figure 5B), probably essential for the structural compactness. Similarly, the mutation into Ser leads to the loss of the entire hydrophobic interactions even if it preserves the ability to make one of the two H-bonds (Figure 5C). Trp80Arg instead modifies the interaction network, losing the possible H-bond with Lys83, preserving the one with Tyr84, and maintaining only the hydrophobic interaction with His39 (Figure 5D). Trp80Leu, judged as having moderate impact by NCI-TCGA, preserves the hydrophobic interaction with His39, loses those with Val81, Tyr84, and Lys78, but creates a novel interaction with Pro41; however, it loses the possibility to create an H-bond with Lys83. In this case, the interaction with the N-loop seems in part preserved, but there is a loss of interaction with the remaining part of the C-terminal helix, giving probably more flexibility to this portion and less compactness as well (Figure 5E). It is important to outline that the “moderate impact” annotation is in agreement with possible effects in terms of structural modifications, as well as with the absence of structural effects but possible modifications in the interaction with receptors or other molecules. Some of the variants without evidence of structural effects are located in regions relevant for protein-protein interactions, as mentioned in Section 3.2.

## 4. Conclusions

Eotaxin-3 protein is closely associated with diseases characterized by eosinophilic inflammation, playing a crucial role in the recruitment and activation of eosinophils in inflamed areas. The observation of the possible effects of amino acid variations on the protein at the structural or functional level makes it possible to highlight what variations could be of particular attention as potential markers of disease conditions. Our study highlighted a structural or functional impact for at least 50 out of 105 amino acid substitutions. An interesting development of this study could concern the effects at a functional level by considering the interaction of eotaxin-3 with other proteins. The substitution of amino acids at the interface may impair the correct interaction with functional consequences. The lack of experimental structures of eotaxin-3 complexed with its interactors prevents the detailed analysis of this aspect. Moreover, the absence of information about the co-occurrence of these variations does not allow for the evaluation of possible additive or compensatory effects. However, this point is of strong relevance at the patient’s level, and the investigations in detecting clinical variants of this gene and protein as double or multiple variants could open interesting scenarios. Moreover, our interest in EoE led us to study this protein, but other proteins could also be studied using the same approach that aims to gain observation on the possible effects of point mutations on proteins in terms of impact on the structure and stability, and possibly function. Therefore, we plan to extend the web application to other proteins related to EoE. The integration of this information may open up new opportunities for the diagnosis and therapy of diseases related to eosinophil-rich inflammation.

## Figures and Tables

**Table 1:** Quality parameters of the 3D structural models of eotaxin-3. The caption “pre-cleaved” indicates the calculation of the parameters on the entire chain of the protein, including the signal peptide not present in the NMR structural model of the secreted form of the protein. The caption “mature” refers to the parameters obtained on models by excluding the signal peptide. Tools and details for the calculation of the parameters are described in the Section 2.

Protein	Z-Score (Pre-Cleaved Form)	Z-Score (Mature Form)	Q-MEAN	RMSD with 1G2S	RAMACHANDRAN PLOT ^a^ (Pre-Cleaved Form) (%)	RAMACHANDRAN PLOT ^a^ (Mature Form) (%)
1G2S (PDB)	/	−5.02	0.620	/	/	69.8–30.2–0–0
AF-Q9Y258-F1 (AlphaFold)	−4.52	−4.93	0.634	1.071	79.8–19–1.2–0	82.5–17.5–0–0
Model_eotaxin-3 (this study)	−4.69	−5.06	0.663	0.164	89.3–10.7–0–0	87.3–12.7–0–0

^a^ The Ramachandran plot analysis by PROCHECK reports the percentage of residues with phi/psi angles in four regions defined as: most preferred–allowed–generously allowed–disallowed.

## Data Availability

The original contributions presented in the study are included in the article/Supplementary Materials; further inquiries can be directed to the corresponding author.

## Supplementary Materials

The following supporting information can be downloaded at: https://www.mdpi.com/article/10.3390/genes15081073/s1, Figure S1: Sequence alignments between eotaxin-3 and other chemokines; Table S1: Summary report; The complete summary of the impact for each variant.
