# Supplementary material for: Bioinformatics Study on Site-Specific Variations of Eotaxin-3, a Key Chemokine in Eosinophilic Esophagitis (EoE)
Source: Genes (Basel). 2024 Aug 14;15(8):1073. doi: 10.3390/genes15081073 (PMC11354214; doi:10.3390/genes15081073)
Supplement: Supplementary file 1 [file genes-15-01073-s001.zip › Supplementary_Figure_S1.pdf]

A

6WWZ_2|Chain B[auth C]|C-C motif chemokine 20|Homo sapiens (9606)
Sequence ID: Query_5514566 Length: 70 Number of Matches: 1
Range 1: 6 to 63

Score	Expect	Method	Identities	Positives	Gaps	Frame
38.9 bits(89)	5e-10()	Compositional matrix adjust.	17/58(29%)	31/58(53%)	2/58(3%)	

```
Query  33  CCFQYSHKPLPWTWVRSY--EFTSNSCSQRAVIFTTKRGKKVCTHPRKKWVQKYISLL  88
           CC  Y+ + L   ++  +  +  +  C   A+IF TK+   VC +P++ WV+  + LL
Sbjct  6   CCLGYTDRILHPKFIVGFTRQLANEGCDINAIIFHTKKKLSVCANPKQTWVKYIVRLL  63
```

Rmsd: 1.686

B

8JPS_2|Chains B[auth C], D|C-C motif chemokine 7|Homo sapiens (9606)
Sequence ID: Query_5514569 Length: 63 Number of Matches: 1
Range 1: 1 to 60

Score	Expect	Method	Identities	Positives	Gaps	Frame
52.0 bits(123)	3e-15()	Compositional matrix adjust.	25/60(42%)	39/60(65%)	1/60(1%)	

```
Query  30  SKTCCFQYSHKPLPWTWVRSYEFTSNS-CSQRAVIFTTKRGKKVCTHPRKKWVQKYISLL  88
           S TCC+++ +K +P   + SY  T++S C + AVIF TK  K++C  P +KWVQ ++  L
Sbjct  1   STTCCYRFINKKIPKQRLESYRRTTSSHCPREAVIFKTKLDKEICADPTQKWVQDFMKHL  60
```

Rmsd: 1.677

C

7VL9_4|Chain D[auth L]|CCL15(26-92)|Homo sapiens (9606)
Sequence ID: Query_5514567 Length: 76 Number of Matches: 1
Range 1: 3 to 63

Score	Expect	Method	Identities	Positives	Gaps	Frame
48.1 bits(113)	1e-13()	Compositional matrix adjust.	23/61(38%)	35/61(57%)	0/61(0%)	

```
Query  29  ISKTCCFQYSHKPLPWTWVRSYEFTSNSCSQRAVIFTTKRGKKVCTHPRKKWVQKYISLL  88
            +  CC  Y  + +P + ++SY  TS+ CS+  VIF TK+G++VC  P    VQ  +  L
Sbjct  3   FAADCCTSYISQSIPCSLMKSYFETSSECSKPGVIFLTKKGRQVCAKPSGPGVQDCMKKL  62

Query  89  K  89
           K
Sbjct  63  K  63
```

Rmsd: 1.104

D

7XA3_2|Chain B[auth L]|C-C motif chemokine 2|Homo sapiens (9606)
Sequence ID: Query_5514568 Length: 69 Number of Matches: 1
Range 1: 10 to 67

Score	Expect	Method	Identities	Positives	Gaps	Frame
46.6 bits(109)	5e-13()	Compositional matrix adjust.	21/58(36%)	35/58(60%)	1/58(1%)	

```
Query  32  TCCFQYSHKPLPWTWVRSYE-FTSNSCSQRAVIFTTKRGKKVCTHPRKKWVQKYISLL  88
           TCC+ ++++ +    + SY   TS+ C + AVIF T   K++C  P++KWVQ  +  L
Sbjct  10  TCCYNFTNRKISVQRLASYRRITSSKCPKEAVIFKTIVAKEICADPKQKWVQDSMDHL  67
```

Rmsd: 1.544

Supplementary Figure S1: *Sequence alignments between eotaxin-3 and other chemokines.*

In panel A: the sequence alignment between eotaxin-3 and CCL20 corresponding to the chain C in the 3D structure of the complex with PDB code: 6WWZ.

In panel B: the sequence alignment between eotaxin-3 and CCL7 corresponding to the chain C in the 3D structure of the complex with PDB code: 8JPS.

In panel C: the sequence alignment between eotaxin-3 and CCL15 corresponding to the chain L in the 3D structure of the complex with PDB code: 7VL9.

In panel D: the sequence alignment between eotaxin-3 and CCL2 corresponding to the chain L in the 3D structure of the complex with PDB code: 7XA3.

For all the alignments, the RMSD calculated between the crystallographic 3-D structure and our eotaxin-3 model is reported.
