# Supplementary material for: Bioinformatics Study on Site-Specific Variations of Eotaxin-3, a Key Chemokine in Eosinophilic Esophagitis (EoE)
Source: Genes (Basel). 2024 Aug 14;15(8):1073. doi: 10.3390/genes15081073 (PMC11354214; doi:10.3390/genes15081073)
Supplement: Supplementary file 1 [file genes-15-01073-s001.zip › Supplementary_Table_S1.pdf]

Supplementary Table S1: Summary report. The complete summary of the impact for each variant at the level of the secondary structure, the solvent accessibility, the stability, the H-bonds, and the salt bridges interaction is reported.

Variant	Residue	Exon	Codon	Amino Acid	Secondary Structure	Solvent Accessibility	Predicted Stability	H-bonds	Salt Bridges
M2T	2:A	1	ATG→ACG	MET→THR	Not Affected	Not Affected	Not Definable	Not Affected	Not Affected
M2V	2:A	1	ATG→GTG	MET→VAL	Not Affected	Not Affected	Not Definable	Not Affected	Not Affected
M2I	2:A	1	ATG→ATA	MET→ILE	Not Affected	Not Affected	Not Definable	Not Affected	Not Affected
G3S	3:A	1	GGC→AGC	GLY→SER	Not Affected	Not Affected	Not Definable	Affected	Not Affected
G3A	3:A	1	GGC→GCC	GLY→ALA	Not Affected	Not Affected	Not Definable	Not Affected	Not Affected
L4I	4:A	1	CTC→ATC	LEU→ILE	Not Affected	Not Affected	Not Definable	Not Affected	Not Affected
L6F	6:A	1	TTG→TTT	LEU→PHE	Not Affected	Not Affected	Not Definable	Not Affected	Not Affected
L6S	6:A	1	TTG→TCG	LEU→SER	Not Affected	Not Affected	Not Definable	Affected	Not Affected
L6V	6:A	1	TTG→GTG	LEU→VAL	Not Affected	Not Affected	Not Definable	Not Affected	Not Affected
L11F	11:A	1	CTC→TTC	LEU→PHE	Not Affected	Not Affected	Not Definable	Affected	Not Affected
L12P	12:A	1	CTG→CCG	LEU→PRO	Not Affected	Not Affected	Not Definable	Affected	Not Affected
L12M	12:A	1	CTG→ATG	LEU→MET	Not Affected	Not Affected	Not Definable	Not Affected	Not Affected
L15H	15:A	1	CTC→CAC	LEU→HIS	Affected	Not Affected	Not Definable	Affected	Not Affected
L16P	16:A	1	CTG→CCG	LEU→PRO	Affected	Not Affected	Not Definable	Affected	Not Affected
L16M	16:A	1	CTG→ATG	LEU→MET	Affected	Not Affected	Not Definable	Not Affected	Not Affected
S17T	17:A	1	AGT→ACT	SER→THR	Not Affected	Not Affected	Not Definable	Affected	Not Affected

S17N	17:A	1	AGT→AAT	SER→ASN	Not Affected	Not Affected	Not Definable	Not Affected	Not Affected
S17I	17:A	1	AGT→ATT	SER→ILE	Not Affected	Not Affected	Not Definable	Not Affected	Not Affected
L18R	18:A	1	CTC→CGC	LEU→ARG	Not Affected	Not Affected	Not Definable	Not Affected	Not Affected
H19Y	19:A	1	CAC→TAC	HIS→TYR	Not Affected	Not Affected	Not Definable	Not Affected	Not Affected
H19Q	19:A	1	CAC→CAG	HIS→GLN	Not Affected	Not Affected	Not Definable	Not Affected	Not Affected
L20F	20:A	1	CTT→TTT	LEU→PHE	Not Affected	Not Affected	Not Definable	Not Affected	Not Affected
A23P	23:A	1	GCC→CCC	ALA→PRO	Not Affected	Not Affected	Not Definable	Not Affected	Not Affected
A23T	23:A	1	GCC→ACC	ALA→THR	Not Affected	Not Affected	Not Definable	Not Affected	Not Affected
A23D	23:A	1	GCC→GAC	ALA→ASP	Not Affected	Not Affected	Not Definable	Not Affected	Not Affected
T24R	24:A	1	ACA→AGA	THR→ARG	Not Affected	Not Affected	Not Definable	Not Affected	Not Affected
T24I	24:A	1	ACA→ATA	THR→ILE	Not Affected	Not Affected	Not Definable	Not Affected	Not Affected
R25C	25:A	1/2	CGT→TGT	ARG→CYS	Not Affected	Not Affected	Not Definable	Not Affected	Not Affected
G26W	26:A	2	GGG→TGG	GLY→TRP	Not Affected	Not Affected	Not Definable	Not Affected	Not Affected
S27N	27:A	2	AGT→AAT	SER→ASN	Not Affected	Not Affected	Not Definable	Not Affected	Not Affected
S27I	27:A	2	AGT→ATT	SER→ILE	Not Affected	Not Affected	Not Definable	Not Affected	Not Affected
D28N	28:A	2	GAC→AAC	ASP→ASN	Not Affected	Not Affected	Not Definable	Not Affected	Not Affected
I29M	29:A	2	ATA→ATG	ILE→MET	Not Affected	Not Affected	Not Definable	Not Affected	Not Affected

S30F	30:A	2	TCC→TTC	SER→PHE	Not Affected	Affected	Not Definable	Not Affected	Not Affected
S30P	30:A	2	TCC→CCC	SER→PRO	Not Affected	Not Affected	Not Definable	Not Affected	Not Affected
S30Y	30:A	2	TCC→TAC	SER→TYR	Not Affected	Affected	Not Definable	Not Affected	Not Affected
S30C	30:A	2	TCC→TGC	SER→CYS	Not Affected	Affected	Not Definable	Not Affected	Not Affected
C33Y	33:A	2	TGC→TAC	CYS→TYR	Not Affected	Not Affected	Not Definable	Not Affected	Not Affected
C34F	34:A	2	TGC→TTC	CYS→PHE	Not Affected	Not Affected	Less Stable	Not Affected	Not Affected
C34S	34:A	2	TGC→TCC	CYS→SER	Not Affected	Not Affected	Less Stable	Affected	Not Affected
C34Y	34:A	2	TGC→TAC	CYS→TYR	Not Affected	Affected	Less Stable	Affected	Not Affected
C34R	34:A	2	TGC→CGC	CYS→ARG	Not Affected	Affected	Less Stable	Affected	Not Affected
Y37H	37:A	2	TAC→CAC	TYR→HIS	Not Affected	Not Affected	Not Definable	Not Affected	Not Affected
S38R	38:A	2	AGC→AGA	SER→ARG	Not Affected	Not Affected	Not Definable	Not Affected	Not Affected
H39Y	39:A	2	CAC→TAC	HIS→TYR	Not Affected	Not Affected	Not Definable	Not Affected	Not Affected
H39Q	39:A	2	CAC→CAA	HIS→GLN	Not Affected	Not Affected	Not Definable	Not Affected	Not Affected
K40R	40:A	2	AAG→AGG	LYS→ARG	Not Affected	Not Affected	Not Definable	Not Affected	Not Affected
K40N	40:A	2	AAG→ACC	LYS→ASN	Not Affected	Not Affected	Not Definable	Not Affected	Not Affected
P41T	41:A	2	CCC→ACC	PRO→THR	Not Affected	Not Affected	Not Definable	Not Affected	Not Affected
L42I	42:A	2	CTT→ATT	LEU→ILE	Not Affected	Not Affected	Not Definable	Not Affected	Not Affected
P43S	43:A	2	CCC→TCC	PRO→SER	Not Affected	Not Affected	Less Stable	Affected	Not Affected

P43R	43:A	2	CCC→CGC	PRO→ARG	Not Affected	Not Affected	Not Definable	Not Affected	Not Affected
W44R	44:A	2	TGG→AGG	TRP→ARG	Not Affected	Not Affected	Not Definable	Not Affected	Not Affected
W44C	44:A	2	TGG→TGC	TRP→CYS	Not Affected	Not Affected	Less Stable	Not Affected	Not Affected
T45S	45:A	2	ACC→AGC	THR→SER	Not Affected	Not Affected	Not Definable	Not Affected	Not Affected
T45I	45:A	2	ACC→ATC	THR→ILE	Not Affected	Not Affected	Not Definable	Not Affected	Not Affected
V47A	47:A	2	GTG→GCG	VAL→ALA	Not Affected	Affected	Less Stable	Not Affected	Not Affected
R48Q	48:A	2	CGA→CAA	ARG→GLN	Not Affected	Affected	Not Definable	Not Affected	Not Affected
S49T	49:A	2	AGC→ACC	SER→THR	Not Affected	Not Affected	Not Definable	Not Affected	Not Affected
S49R	49:A	2	AGC→AGA	SER→ARG	Not Affected	Not Affected	Not Definable	Affected	Not Affected
Y50D	50:A	2	TAT→GAT	TYR→ASP	Not Affected	Not Affected	Less Stable	Not Affected	Not Affected
T53S	53:A	2	ACC→AGC	THR→SER	Not Affected	Not Affected	Less Stable	Affected	Not Affected
S56R	56:A	2	AGC→AGA	SER→ARG	Not Affected	Not Affected	Not Definable	Affected	Not Affected
S58T	58:A	2	TCC→ACC	SER→THR	Not Affected	Not Affected	Not Definable	Not Affected	Not Affected
S58C	58:A	2	TCC→TGC	SER→CYS	Not Affected	Not Affected	Not Definable	Not Affected	Not Affected
R60W	60:A	2	CGG→TGG	ARG→TRP	Not Affected	Not Affected	Not Definable	Not Affected	Not Affected
R60Q	60:A	2	CGG→CAG	ARG→GLN	Not Affected	Not Affected	Not Definable	Not Affected	Not Affected
A61T	61:A	2	GCT→ACT	ALA→THR	Not Affected	Not Affected	Less Stable	Not Affected	Not Affected

A61V	61:A	2	GCT→GTT	ALA→VAL	Not Affected	Not Affected	Not Definable	Not Affected	Not Affected
V62G	62:A	2	GTG→GGG	VAL→GLY	Not Affected	Not Affected	Less Stable	Affected	Not Affected
I63R	63:A	2/3	ATA→AGA	ILE→ARG	Not Affected	Not Affected	Less Stable	Affected	Not Affected
F64C	64:A	3	TTC→TGC	PHE→CYS	Not Affected	Not Affected	Less Stable	Not Affected	Not Affected
F64L	64:A	3	TTC→CTC	PHE→LEU	Not Affected	Not Affected	Less Stable	Not Affected	Not Affected
T65I	65:A	3	ACT→ATT	THR→ILE	Not Affected	Not Affected	Not Definable	Not Affected	Not Affected
T66A	66:A	3	ACC→GCC	THR→ALA	Not Affected	Not Affected	Less Stable	Affected	Not Affected
T66I	66:A	3	ACC→ATC	THR→ILE	Not Affected	Not Affected	Not Definable	Affected	Not Affected
R68S	68:A	3	AGA→AGT	ARG→SER	Not Affected	Not Affected	Not Definable	Affected	Not Affected
G69S	69:A	3	GGC→AGC	GLY→SER	Not Affected	Not Affected	Not Definable	Not Affected	Not Affected
V72I	72:A	3	GTC→ATC	VAL→ILE	Not Affected	Not Affected	Not Definable	Not Affected	Not Affected
C73Y	73:A	3	TGT→TAT	CYS→TYR	Not Affected	Affected	Not Definable	Not Affected	Not Affected
T74N	74:A	3	ACC→AAC	THR→ASN	Not Affected	Not Affected	Less Stable	Affected	Not Affected
T74I	74:A	3	ACC→ATC	THR→ILE	Not Affected	Not Affected	Not Definable	Affected	Not Affected
P76S	76:A	3	CCA→TCA	PRO→SER	Not Affected	Not Affected	Not Definable	Not Affected	Not Affected
P76T	76:A	3	CCA→ACA	PRO→THR	Not Affected	Not Affected	Not Definable	Not Affected	Not Affected
R77G	77:A	3	AGG→GGG	ARG→GLY	Not Affected	Not Affected	Not Definable	Not Affected	Not Affected
R77K	77:A	3	AGG→AAG	ARG→LYS	Not Affected	Not Affected	Not Definable	Not Affected	Not Affected

K78E	78:A	3	AAA→GAA	LYS→GLU	Not Affected	Not Affected	Not Definable	Affected	Affected
K79T	79:A	3	AAA→ACA	LYS→THR	Not Affected	Not Affected	Not Definable	Affected	Not Affected
W80S	80:A	3	TGG→TCG	TRP→SER	Not Affected	Not Affected	Less Stable	Affected	Not Affected
W80R	80:A	3	TGG→CGG	TRP→ARG	Not Affected	Affected	Less Stable	Affected	Not Affected
W80G	80:A	3	TGG→GGG	TRP→GLY	Not Affected	Affected	Less Stable	Affected	Not Affected
W80L	80:A	3	TGG→TTG	TRP→LEU	Not Affected	Not Affected	Less Stable	Affected	Not Affected
V81M	81:A	3	GTG→ATG	VAL→MET	Not Affected	Not Affected	Not Definable	Not Affected	Not Affected
Q82P	82:A	3	CAA→CCA	GLN→PRO	Not Affected	Not Affected	Not Definable	Affected	Not Affected
K83T	83:A	3	AAA→ACA	LYS→THR	Not Affected	Not Affected	Not Definable	Affected	Not Affected
K83R	83:A	3	AAA→AGA	LYS→ARG	Not Affected	Not Affected	Not Definable	Not Affected	Not Affected
Y84N	84:A	3	TAC→AAC	TYR→ASN	Not Affected	Not Affected	Less Stable	Affected	Not Affected
I85T	85:A	3	ATT→ACT	ILE→THR	Not Affected	Not Affected	Less Stable	Not Affected	Not Affected
I85V	85:A	3	ATT→GTT	ILE→VAL	Not Affected	Not Affected	Not Definable	Not Affected	Not Affected
S86F	86:A	3	TCT→TTT	SER→PHE	Not Affected	Not Affected	Not Definable	Not Affected	Not Affected
S86Y	86:A	3	TCT→TAT	SER→TYR	Not Affected	Not Affected	Not Definable	Not Affected	Not Affected
P91L	91:A	3	CCG→CTG	PRO→LEU	Not Affected	Not Affected	Not Definable	Not Affected	Not Affected
K92T	92:A	3	AAA→ACA	LYS→THR	Not Affected	Not Affected	Not Definable	Not Affected	Not Affected

Q93H	93:A	3	CAA→CAC	GLN→HIS	Not Affected	Not Affected	Not Definable	Not Affected	Not Affected
L94V	94:A	3	TTG→GTG	LEU→VAL	Not Affected	Not Affected	Not Definable	Not Affected	Not Affected
